# Clinical and radiographic periodontal status in hypertensive patients with or without obstructive sleep apnea 10 years after diagnosis and CPAP initiation

**DOI:** 10.1002/cre2.859

**Published:** 2024-03-03

**Authors:** Christine Kvarnvik, Hanna Ahonen, Henrik Jansson, Anders Broström, Malin Stensson, Shariel Sayardoust

**Affiliations:** ^1^ School of Health and Welfare, Center for Oral Health Jönköping University Jönköping Sweden; ^2^ Department of Periodontology Postgraduate Dental Education, The Institute of Odontology, Region Jönköping County Jönköping Sweden; ^3^ Department of Odontology and Oral Health Sciences Jönköping University Jönköping Sweden; ^4^ Folktandvården Skåne Lund Sweden; ^5^ School of Health and Welfare Jönköping University Jönköping Sweden; ^6^ Department of Clinical Neurophysiology Linköping University Hospital Linköping Sweden; ^7^ Department of Health and Caring Sciences, Faculty of Health and Social Sciences Western Norway University of Applied Sciences Bergen Norway; ^8^ Department of Biomedical and Clinical Sciences Linköping University Linköping Sweden; ^9^ Center for Oral Rehabilitation Linköping Sweden

**Keywords:** continuous positive airway pressure, hypertension, obstructive sleep apnea, periodontal health

## Abstract

**Objectives:**

Through inflammation and hyposalivation, obstructive sleep apnea (OSA) is suggested to affect periodontal status over time. Our aim was to compare the clinical and radiographic periodontal status of hypertensive patients with or without long‐term presence of OSA, treated or untreated with continuous positive airway pressure treatment (CPAP).

**Materials and Methods:**

In 2007–2009, a screening for OSA was conducted among 394 hypertensive primary care patients. Polygraphy was used to create three groups: no OSA, non‐CPAP, or adherent CPAP based on the apnea hypopnea index (AHI). After 10 years, a cross‐sectional sleep and periodontal examination including a clinical and radiographic examination, a questionnaire, and a matrix metalloproteinase‐8 (MMP‐8) chair‐side test was conducted. Based on levels of alveolar bone, bleeding on probing (BoP), and probing pocket depth (PPD), patients were categorized into four periodontal stages: periodontal health/gingivitis and three periodontal disease stages. Periodontal status and periodontal stages were compared between the OSA (*n* = 49), non‐CPAP (*n* = 38), or adherent CPAP (*n* = 34) groups.

**Results:**

The 121 patients (53% women) had a median age of 71 years. No differences were seen between the OSA groups regarding median number of teeth (*p* = .061), teeth/implants, (*p* = .107), plaque index (*p* = .245), BoP (*p* = .848), PPD ≥ 4 mm (*p* = .561), PPD ≥ 6 mm (*p* = .630), presence of MMP‐8 (*p* = .693) except for bone loss (*p* = .011). Among patients with stage periodontal health/gingivitis a significant difference was seen, as 70% of those were categorized as no OSA, 20% as non‐CPAP, and 10% as adherent CPAP (*p* = .029). Differences were not seen in periodontal disease stages.

**Conclusions:**

Hypertensive patients with obstructive sleep apnea (OSA) did not have an adverse clinical periodontal status compared to patients without OSA. However, when combining radiographic and clinical status into periodontal stages, patients without OSA more frequently exhibited periodontal health or gingivitis compared to patients without OSA, regardless of CPAP treatment.

## 1 INTRODUCTION

Periodontal health is defined as the absence of clinical inflammation and periodontal disease, which allows normal oral function (Chapple et al., 2018; Tonetti et al., 2018). In contrast, periodontitis is defined as a multifactorial inflammatory disease with a chronic destructive inflammatory response which is also associated with dysbiosis in the biofilm surrounding the teeth, and if untreated leads to periodontal attachment and bone loss (Papapanou et al., 2018). A balance between the host response and modifying and predisposing risk factors prevents progression into periodontal disease (Lang & Bartold, 2018). A relatively new suggested risk factor for periodontitis is obstructive sleep apnea (OSA), but whether there is a causal relationship or not, due to systemic inflammation and hyposalivation, is debated (Dort, 2017). This area of research is still in its infancy, with most existing studies being observational, such as the pioneering work by Gunaratnam et al. (2009) which reported a higher incidence of periodontitis in OSA patients, a finding echoed in subsequent research (Zhu et al., 2023). Individuals with periodontitis have also been reported to have a higher prevalence of severe OSA (Arango Jimenez et al., 2023). As approximately 769 million people suffer from severe periodontal disease globally (Bernabe et al., 2020) this could affect a large number of people. Periodontitis alone is one of the main reasons why oral diseases persist as a major public health challenge (Kassebaum et al., 2017). Moreover, there is also an association between periodontal disease and other common systemic diseases such as diabetes (Graziani et al., 2018; Nascimento et al., 2018; Sanz et al., 2018) and cardiovascular disease (CVD) (Ryden et al., 2016; Sanz et al., 2020). Clinical examination and radiographs are still the golden standard as periodontal diagnostic tools, but recently biomarkers in saliva such as matrix metalloproteinases‐8 (MMP‐8), MMP‐9, interleukin‐1 β, and IL‐6 have also been proposed for detection of periodontal disease (Arias‐Bujanda et al., 2020).

OSA is characterized by partial (hypopnea) or total (apnea) obstruction in the pharynx during sleep (Young et al., 2002), which leads to sleep disturbance and hypoxemia (Dewan et al., 2015). It is a highly prevalent disorder (Gottlieb & Punjabi, 2020; Senaratna et al., 2017) and increases with age (Senaratna et al., 2017). There is a relationship between OSA and hypertension/CVD which often goes both ways (Javaheri et al., 2017). Continuous positive airway pressure treatment (CPAP) is a common treatment of OSA but side effects, such as dry mouth are common (Ahonen et al., 2022; Ulander et al., 2014). Apnoic breathing‐related events cause desaturation, oxidative stress, sympathetic nerve activity, metabolic, vascular, and endothelial dysfunction and also support underlying systemic inflammation (Dewan et al., 2015; Javaheri et al., 2017). Combined, this could lead to elevated hypertension, heart failure, and CVD (Javaheri et al., 2017; Virani et al., 2021). It has also been shown that OSA patients have an elevated intracellular production of reactive oxygen species (ROS) whereas CPAP treatment downregulated the production of ROS (Dewan et al., 2015). The imbalance of ROS and the antioxidant response can cause injuries that can affect the periodontal tissues negatively and increase the risk for periodontal disease (Liu et al., 2017; Sczepanik et al., 2020).

A bidirectional association between OSA and periodontitis is suggested but more research is warranted (Al‐Jewair et al., 2020). Periodontitis and OSA share risk factors/risk indicators as well as theories linking the two conditions together, but the pathophysiology and cause‐effect between OSA and periodontitis is still unclear (Lembo et al., 2021). However, the earlier described pathophysiological mechanisms due to oxidative stress and increased systemic inflammation by OSA are hypothesized to have a negative impact on other parts of the body (Dewan et al., 2015) such as the periodontal tissues (Gamsiz‐Isik et al., 2017; Lavie, 2015; Téllez‐Corral et al., 2023). Due to sleep disruption, levels of inflammation markers for example, interleukin‐6 (IL‐6) and C reactive protein in serum (Irwin et al., 2016) and IL‐6 in saliva are increased (Nizam et al., 2016). OSA may increase the risk of periodontal disease due to increased inflammatory cytokines (Téllez‐Corral et al., 2023). Another suggested linkage between OSA and periodontitis is hyposalivation (Lembo et al., 2021; Seo et al., 2013) and an altered microbiota caused by mouth breathing (Téllez‐Corral et al., 2022). The potential connection between OSA and periodontitis points to the importance of being aware of the patient's OSA condition and treatment to improve periodontal treatment planning and treatment (Téllez‐Corral et al., 2023). It is important to know if OSA has a negative effect on periodontal health, and if so, whether CPAP treatment with a humidifier has a positive effect on periodontal health, due to diminished systemic inflammation.

Our hypothesis is that patients without OSA are more associated with periodontal health compared to patients with OSA, and that long‐term CPAP treatment could decrease the hypopnea/apnea events and in that way diminish the risk for periodontal disease in patients with OSA. The aim is therefore to compare clinical and radiographic periodontal status in hypertensive individuals with or without the long‐term presence of OSA, treated or not treated with CPAP.

## 2 MATERIALS AND METHODS

### 2.1 Ethics

The study protocol of this cross‐sectional study with a comparative design was approved by the Regional Ethical Board in Linköping (ref.no: 2018/36‐31). A written informed consent agreement was signed by all patients. The study was performed according to the guidelines from the Helsinki Declaration (World Medical Association, 2013). After the examinations, patients were informed both orally and in writing about their status and whether contact with dental care was needed.

### 2.2 Study population

#### 2.2.1 Initial data collection regarding hypertension and OSA

In 2007–2009 a longitudinal study on hypertensive patients was conducted in southern Sweden to investigate a potential relationship between hypertension and OSA (Broström et al., 2012). All patients aged 18–65 years at four primary care centers with hypertension >140/90 mmHg were asked to participate. Patients with dementia, tumor diagnosis, psychiatric diagnosis, short survival prognosis, or difficulty understanding Swedish were excluded. Clinical health measurements such as blood pressure, body mass index, apnea hypopnea index (AHI), and registrations of diseases and medications were made at a sleep clinic by a nurse specialist. Measurements of OSA were conducted with a full night polygraphic recording (Embletta ResMed AB) at home (Collop et al., 2007; Kapur et al., 2017). AHI was categorized as mild OSA (i.e., AHI 5–14.9/h) and moderate/severe OSA (i.e., AHI > 15/h, moderate >30 severe) (Gottlieb & Punjabi, 2020).

In total, 394 patients participated and the results showed that 59% of the hypertensive patients also were diagnosed with OSA (AHI ≥ 5) (i.e., mild OSA 29%, moderate/severe OSA 30%) (Broström et al., 2012). Ten years after the initial study was conducted, the 394 hypertensive patients were invited to participate in a follow‐up study regarding oral health.

### 2.3 10‐year follow‐up examination

**Figure 1:**
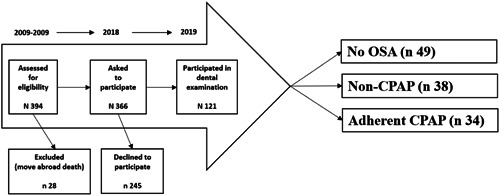
Flow chart of the selection process from baseline 2007–2009 to dental examination 2019 and group selection based on AHI and hypertension and CPAP or no CPAP treatment; Group selection is based on AHI and hypertension and CPAP or no CPAP treatment; no OSA (*n* 49), non‐CPAP (*n* 38) and adherent CPAP (*n* 34).

In the 10‐year follow‐up, 366 patients were invited to participate because 28 patients had migrated abroad or were deceased (Figure 1). The periodontal examination was part of a wider dental examination. Patients answered a questionnaire including self‐reported information regarding age, sex, living conditions, education, working hours, and habits concerning oral health and tobacco use. The periodontal examination included both clinical periodontal status and radiographic status. Registrations of clinical periodontal status included plaque index (PLI) (Ainamo & Bay, 1975), bleeding on probing (BoP), probing pocket depth (PPD), furcation involvement (II, III), and mobility. Registrations were performed using a periodontal probe (Hu‐Friedy) at four sites/teeth (i.e., mesial, buccal, distal, and lingual/palatinal).

Six radiographs (i.e., bitewing) were taken on each patient but if radiographs (<1 year) were registered these were used instead. If the marginal bone was not possible to see with the bitewing, apical radiographs were taken as a complement. Two examiners (the first and second authors) reviewed the radiographs and bone loss was registered on the patient level. Bone loss was defined as predominantly bone level >2 mm from the cemento enamel junction (CEJ) on bitewing or no bone loss predominantly bone level <2 mm from CEJ.

A chair‐side test regarding MMP‐8 was conducted during the examination. All examinations were conducted in the afternoon either at 1 or 3 pm. The nonstimulated saliva was collected for 5 min and analyzed with an MMP‐8 chair‐side test described by Sorsa et al. (2020) with a cut‐off of *n* > 20 ng/mL for a positive test (Alassiri et al., 2018)

The first and second authors (dentist, dental hygienist) underwent calibration training before onset of the data collection, including comparing BoP, PPD, tooth mobility, furcation involvement, and radiographs. The examiners were blinded to the OSA diagnosis until after the completion of the dental examination. In dubious cases, one of the two examiners, the first author, reviewed the radiographs a second time before the group formation. Two pilot examinations on two volunteer participants, who were not related to the study population, were completed before the study started. These individuals represented one person with hypertension and one person with hypertension and CPAP‐treated OSA. These examinations were made by both examiners on two separate occasions, before onset of the data collection.

### 2.4 Group formation

#### 2.4.1 Groups according to OSA

Patients were categorized into three groups of hypertensive patients: no OSA, non‐CPAP, and adherent CPAP (Table 1). Group formation was based on AHI and hypertension, which were collected at the examination in 2007–2009. Patients were categorized as hypertensive if blood pressure (BP) >140/90 mmHg and with OSA if AHI > 5. Groups of hypertensive patients were formed into: no OSA (*n* = 49), diagnosed with OSA non‐CPAP (*n* = 38) and adherent CPAP (*n* = 34).

**Table 1:** Definition of OSA groups and periodontal health stages used in the current study.

A. OSA groups	
No OSA	BP > 140/90 mmHg, AHI < 5
Non‐CPAP	BP > 140/90 mmHg, OSA based on AHI > 5 Non‐CPAP‐adherent, abandoned CPAP, prescribed oral appliances
Adherent CPAP	BP > 140/90 mmHg and OSA based on AHI > 5, CPAP‐adherent use (70% more than 4 h/night)
B. Stages of periodontal health and periodontitis	
Periodontal health/gingivitis	No bone loss and <10% BoP Periodontal (*health*) or BoP≥10% (g*ingivitis*)
Periodontitis with BoP <10%	Bone loss, <10% BoP, may include individuals with 4–5 mm pockets
Mild periodontitis with BoP ≥10%	Bone loss ≥10% BoP, with 4–5 mm pockets
Advanced periodontitis with BoP ≥10%	Bone loss, ≥10% BoP, with 6 mm pockets

Abbreviation: OSA, obstructive sleep apnea.

Objective CPAP adherence was verified by the devices (h/night) and had to be >4 h/night (70% nights/year) to be adherent (Sawyer et al. (2011). A specialist nurse at the sleep medicine clinic analyzed the medical records. The majority of the CPAP users had a device with a humidifier. Patients undergoing treatment with oral appliances were included in the hypertension group diagnosed with OSA.

#### 2.4.2 Groups according to periodontal stages

After the periodontal examination, patients were also divided into four different periodontal health/disease stages: periodontal health/gingivitis, periodontitis with BoP <10%, mild periodontitis with BoP ≥10%, advanced periodontitis with BoP ≥10%, based on the periodontal bone level (bitewing), BoP, and PPD (Table 1). To be defined as bone loss (on patient level) a bone level more than 2 mm from CEJ had to be registered on X‐ray. The bone loss also had to be predominantly registered at X‐ray and not local. The periodontal stages were based on the principle of the staging classifications to place periodontal status into groups from the 2017 World Workshop (Tonetti et al., 2018), but modifications had to be made in accordance with the data. During the examination of clinical periodontal status, all teeth were included, as well as dental implants. In this study, PPD and BoP registrations on dental implants and third molars were excluded for inclusion in periodontal stages groups.

#### 2.4.3 Groups according to OSA groups and periodontal stages

**Figure 2:**
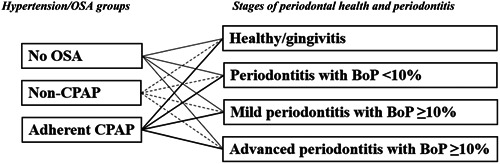
The interplay between OSA groups and periodontal stage groups on patient level, for hypertensive patients. Patients were categorized into one hypertension/OSA group and one periodontal health/disease stage. There were 12 different combinations categorizing individuals according to both criteria of hypertension/OSA group and periodontal stage.

All patients were categorized based on four different periodontal stages and three different groups of OSA, as previously described. This resulted in 12 potential combinations of these subgroups, as illustrated in Figure 2. Patients were categorized into one of these combinations according to the criteria of both OSA and periodontal stage groups.

### 2.5 Statistical methods

Initially, the included variables were analyzed for normality by using both Kolmogorov–Smirnov and Shapiro–Wilks test. As some of the variables showed non‐normal distribution, statistical analysis was performed using a Kruskal–Wallis test. If only one of the normality tests indicated a statistically significant difference, a one way analysis of variance test was used to verify the results. For categorical data, the *χ* ^2^ test was used. A significance level of *α* = .05 was adopted for all analyses, which were conducted using IBM SPSS version 27.

## 3 RESULTS

### 3.1 Demography and health measures and habits

In total, 121 hypertensive individuals (female 53%) with a median age of 71 years (mean age 69 years) participated (age range 49–77 years). Eleven individuals (9%) were diagnosed with diabetes, but no differences were seen among the groups. 25% were former smokers (now ceased) and 5% were current smokers. Table 2 lists the demographic information and Table 3 describes medication, smoking habits and health measures of the OSA groups. No statistically significant differences were seen among groups except for sex (*p* = .048) and AHI (*p* < .001).

**Table 2:** Demographic information regarding hypertension/OSA groups.

		No OSA (*n* 49)	Non‐CPAP (*n* 38)	Adherent CPAP (*n* 34)	*p* Value
Age^a^	Years	71.0 (68.0–73.0)	68.5 (64.0–73.0)	72.5 (69.5–74.3)	.073^c^
Sex^b^	Male	17 (34.7)	19 (50.0)	21 (61.8)	.048^d^
	Female	32 (65.3)	19 (50.0)	13 (38.2)	
Living conditions^b^	Cohabitant	35 (71.4)	27 (71.1)	27 (79.4)	.658^d^
	Living alone	14 (28.6)	11 (28.9)	7 (20.6)	
University/college degree^b^	Yes	21 (42.9)	15 (39.5)	8 (23.5)	.176^d^
	No	28 (57.1)	23 (60.5)	26 (76.5)	
Working hours^b^	Full‐time	4 (8.2)	7 (18.4)	7 (20.6)	.129^d^
	Part‐time	6 (12.2)	7 (18.4)	1 (2.9)	
	Pensioner	39 (79.6)	24 (63.2)	26 (76.5)	
Years at the same dental clinic (years)^b^	0–1	0 (0.0)	2 (5.3)	2 (5.9)	.103^d^
	>1<10	12 (24.5)	15 (39.5)	6 (17.6)	
	≥10	37 (75.5)	21 (55.3)	26 (76.5)	
Toothbrush frequency^b^	1 time/day	2 (4.1)	5 (13.2)	5 (14.7)	.159^d^
	2 times/day	40 (81.6)	25 (65.8)	27 (79.4)	
	>2 times/day	7 (14.3)	8 (21.1)	2 (5.9)	
Interdental cleaning frequency^b^	No	2 (4.1)	0 (0.0)	3 (8.8)	.366^d^
	Occasionally	9 (18.4)	11 (28.9)	4 (11.8)	
	>3 times/week	11 (22.4)	9 (23.7)	9 (26.4)	
	Every day	27 (55.1)	18 (47.4)	18 (52.9)	
Dental visits last 24 months	No time	2 (4.1)	6 (15.8)	3 (8.8)	.375^d^
	1–3 times	36 (73.5)	26 (68.4)	26 (76.5)	
	≥4 times	11 (22.4)	6 (15.8)	5 (14.7)	

*Note*: Self‐reported data was collected in dental examinations 2018–2019 (*N*: 121, no missing). For continuous values, data is presented as median (interquartile range) and data for categorical variables is presented as frequency (percentage).

^a^ Median (interquartile range).

^b^ (%).

^c^ Independent‐samples Kruskal–Wallis test.

^d^ *χ* ^2^ test.

**Table 3:** Health measures and tobacco habits regarding hypertensive patients with or without OSA.

		No OSA (*n* 49)	Non‐CPAP (*n* 38)	Adherent CPAP (*n* 34)	*p* Value
Tobacco^b^	Never	34 (69.4)	21 (55.3)	20 (58.8)	.366^d^
	Smoker	2 (4.1)	2 (5.3)	2 (5.9)	.928^d^
	Former smoker	10 (20.4)	9 (23.7)	11 (32.4)	.456^d^
	Snuff: Present	1 (2.0)	4 (10.5)	1 (2.9)	.159^d^
	Snuff: Past	4 (8.2)	3 (7.9)	1 (2.9)	.596^d^
Health measures	Diabetes^b^	4 (8.2)	4 (10.8)	3 (9.4)	.917^d^
	SBP^a^	135.0 (130.0–152.5)	140.0 (130.0–150.0)	140.0 (120.0–152.5)	.931^c^
	DBP^a^	90.0 (80.0–90.0)	87.5 (80.0–90.0)	85.0 (80.0–90.0)	.657^c^
	AHI^a^	2.30 (0.95–3.65)^2^, ^3^, ***	11.70 (7.80–18.2)^1^, *** ^,^ 3, *	24.10 (17.3–36.98)^1^, *** ^,^ 2, *	<.001^c^
	BMI^a^	27.1 (24.0–29.6)	27.6 (26.0–31.2)	28.9 (26.2–31.2)	.183^c^
Medications^b^	Calcium antagonists	8 (16.3)	8 (21.1)	11 (33.3)	.188^d^
	Beta‐blockers	20 (40.8)	18 (47.4)	17 (51.5)	.618^d^
	ACE inhibitors	32 (65.3)	23 (60.5)	16 (48.5)	.308^d^
	Diuretics	13 (26.5)	12 (31.6)	11 (33.3)	.779^d^
	Hypnotics	3 (6.1)	2 (5.3)	0 (0.0)	.364^d^
	Antidepressants	3 (6.1)	3 (7.9)	4 (12.1)	.624^d^

*Note*: Data from baseline examination 2007–2009: Diabetes, blood pressure (systolic/diastolic), AHI, BMI, medications. Data from dental examinations 2018–2019: Tobacco use. Data is presented as median (interquartile range) for continuous values and as frequency (percentage) for categorical variables. *N* 121 individuals: Diabetes. Missing (total *n* = 3) (two missing in group‐adherent CPAP and one missing in non‐CPAP). Blood pressure (SBP, DBP), BMI, medication missing: Total *n* = 1 (in subgroup‐adherent CPAP).

Abbreviations: AHI, apnea hypopnea index; BMI, dody mass index; DBP, diastolic blood pressure; SBP, systolic blood pressure. Significant values have been adjusted by the Bonferroni correction for multiple tests.

^a^ Median (interquartile range).

^b^ (%).

^c^ Independent‐samples Kruskal–Wallis test.

^d^ *χ* ^2^ test.

^1^ No OSA

^2^ Non‐CPAP.

^3^ Adherent CPAP.

*** *p* < 0.001

* *p* < 0.05.

### 3.2 Periodontal outcome measures

No statistically significant differences were observed regarding the number of teeth, number of teeth with implants, PLI, BoP, median number of PPD ≥ 4 mm, or PPD ≥ 6 mm between the hypertensive patients, no OSA, non‐CPAP, and adherent CPAP (Table 4). In total, 34 participants had furcation involvement grade II (i.e., minimum 1–maximum 9), 7 participants had furcation involvement grade III (i.e., minimum 1–maximum 8), and 6 participants had tooth mobility grade 2 (i.e., minimum 1–maximum 3) at least for 1 tooth. None of the participants had tooth mobility grade 3. No statistically significant differences were seen regarding furcation grade II (*p* = .186), furcation grade III (*p* = .192), or mobility 2 (*p* = .538) between hypertension no OSA, non‐CPAP, and adherent CPAP. The presence of MMP‐8 (chair‐side test) was identified in 59 individuals (49%) and no statistically significant differences were seen between the no OSA, non‐CPAP, and adherent CPAP groups (Table 4). A significant difference was seen regarding predominantly bone loss defined as more than 2 mm from CEJ on X‐ray (*p* = .011). Predominantly no bone loss was registered in 14 patients (70%) with hypertension, 4 patients with non‐CPAP (20%), and 2 patients adherent CPAP (10%). 35 patients with hypertension (34.7%), 34 non‐CPAP (33.7%), and 32 individuals (31.7%) adherent CPAP had predominantly bone loss.

**Table 4:** Periodontal parameters as single outcome measurements in hypertensive patients with or without OSA.

		No OSA (*n* 49)	Non‐CPAP (*n* 38)	Adherent CPAP (*n* 34)	*p* Value
No. of teeth^a^		26.0 (24.0–28.0)	27.0 (24.0–29.0)	25.0 (20.0–28.0)	.061^b^
No. of teeth and implants^a^		26.0 (24.0–28.0)	27.0 (24.0–29.0)	25.5 (21.8–28.0)	.107^b^
PLI in %^a^		26.0 (7.49–55.7)	21.2 (9.65–40.5)	15.9 (2.29–49.1)	.245^b^
BoP in %^a^		12.0 (3.94–19.5)	13.1 (3.30–27.1)	12.3 (4.40–23.1)	.848^b^
No. of surfaces with periodontal pockets	≥4 mm^a^	10.0 (2.50–20.50)	14.0 (6.00–25.75)	8.00 (3.75–27.25)	.561^b^
	≥6 mm^a^	0.00 (0.00–3.00)	0.00 (0.00–2.25)	0.50 (0.00–6.00)	.630^b^
MMP‐8	YES^c^	22 (44.9)	20 (52.6)	17 (52.1)	.693^d^
	NO^c^	27 (55.1)	18 (47.4)	15 (49.9)	

*Note*: Data from dental examinations 2018–2019: Periodontal parameters in 121 hypertensive individuals with or without OSA and with or without CPAP treatment. Third molar and implants are included. Presence of MMP‐8 in saliva (chair‐side test). Number of teeth, teeth and implants, PLI, BoP and number of surfaces with periodontal pockets: No missing. MMP‐8: Missing 2 (in subgroup‐adherent CPAP).

Abbreviations: OSA, obstructive sleep apnea; PLI, plaque index.

^a^ *N* median (p‐25‐p75).

^b^ Independent‐samples Kruskal–Wallis test.

^c^ (%).

^d^ *χ2* test.

### 3.3 The interplay between periodontal stages and OSA

Patients were categorized based on periodontal staging as follows: periodontal healthy/gingivitis (*n* = 20), periodontitis BoP <10% (*n* = 28), mild periodontitis BoP ≥10% (*n* = 32), and advanced periodontitis BoP ≥10% (*n* = 41) (Table 5). When integrating radiographic and clinical status (PPD and BoP) and forming groups of periodontal stages a statistically significant difference was seen among periodontal stages in combination with patients with no OSA, non‐CPAP, and adherent CPAP (Figure 2). Among individuals (*n* = 20) categorized as periodontal healthy/gingivitis, 70% had no OSA, 20% had non‐CPAP and 10% had OSA adherent to CPAP (*p* = .029) (Table 5). In the same group (i.e., periodontal healthy/gingivitis) post hoc tests revealed statistically significant differences among groups with no OSA and non‐CPAP (*p* = .0015) and groups with no OSA and adherent CPAP (*p* < .0001) (Table 5). Among individuals categorized with different stages of periodontal disease, no statistically significant differences were observed regarding distribution between the OSA, non‐CPAP, and adherent CPAP groups (Table 5).

**Table 5:** Comparison between frequency of periodontal stages groups and OSA groups in hypertensive patients.

	No OSA	Non‐CPAP	Adherent CPAP	*p* Value
Healthy/gingivitis (*n* 20)	14 (70.0)^2^, ** ^,^ ^3^, ***	4 (20.0)^1^, **	2 (10.0)^1^, ***	.029^a^
Periodontitis with BoP < 10% (*n* 28)	7 (25.0)	9 (32.1)	12 (42.9)	
Mild periodontitis with BoP ≥ 10% (*n* 32)	11 (34.4)	14 (43.8)	7 (21.9)	
Advanced periodontitis with BoP ≥ 10% (*n* 41)	17 (41.5)	11 (26.8)	13 (31.7)	

*Note*: The 121 individuals divided into groups no OSA, non‐CPAP and adherent CPAP compared with periodontal health stages: Healthy/gingivitis, periodontitis with BoP < 10%, mild periodontitis ≥10% and advanced periodontitis ≥10%. Third molar and implants are not included when forming periodontal stage groups.

Abbreviation: OSA, obstructive sleep apnea.

^a^ *χ* ^2^ test.

^1^ No OSA.

^2^ Non‐CPAP.

^3^ Adherent CPAP.

*** <0.001.

** <0.01.

## 4 DISCUSSION

### 4.1 Results

The aim was to explore the link between clinical and radiographic periodontal status in hypertensive individuals, considering their OSA status 10 years after CPAP treatment had been initiated. Intriguingly, no significant differences emerged at a group level in clinical status between individuals without OSA, those with untreated OSA, and CPAP‐adherent patients. However, a notable observation was that individuals without an OSA diagnosis more frequently exhibited periodontal health or gingivitis at an individual level, a pattern that remained consistent irrespective of compliant CPAP treatment use. This finding underscores a potential association between OSA absence and better periodontal health, challenging our initial hypothesis that long‐term CPAP treatment could mitigate periodontal disease progression.

To contextualize our findings, it is essential to compare them with existing research. Our results resonate with those of Loke et al. (2015) who also observed no significant group‐level differences in clinical periodontal status (e.g., bleeding and PPD) among different OSA categories. In contrast, Gamsiz‐Isik et al. (2017) found significantly higher clinical parameters (i.e., PLI, BoP, PPD, CAL) in OSA patients compared to non‐OSA controls. A similar result was presented by Seo et al. (2013) in terms of gingival bleeding, PPD, and CAL. The variation in these results may stem from how OSA groups were classified. In our study, as well as in Loke et al. (2015), participants were categorized into specific OSA subgroups, unlike other studies where participants were broadly classified as having OSA or not (Gamsiz‐Isik et al., 2017; Gunaratnam et al., 2009; Seo et al., 2013). This difference in classification is highlighted by (Verhelst et al., 2022) who, upon dividing OSA groups into three risk subgroups in relation to periodontitis, found no association in the separate groups but did observe an association when combining the low and intermediate risk groups. This approach of dividing OSA and periodontal disease into subgroups, while informative, may have reduced the statistical power of our study. It may also explain the observed significant differences at the individual level but not at the group level. Our study's unique design, differentiating between CPAP‐adherent and nonadherent OSA patients, was crucial, especially considering the common occurrence of nonadherence to CPAP treatment within the first 6 months (Rotenberg et al., 2016). This design, taking into account the severity of OSA and the hypothesized benefits of CPAP treatment with a humidifier on periodontal health, was essential despite the potential reduction in statistical power.

An additional factor influencing our results was the diversity within the non‐OSA group, which included both patients with good periodontal health and those with advanced periodontal disease. This variability could have masked significant differences in median clinical periodontal status values between OSA and non‐OSA groups. Consequently, while significant differences in clinical status were noted at an individual level, they were not evident at the group level. Similarly, no differences in MMP‐8 levels were observed across OSA groups, which might be attributed to the reasons discussed above and possibly the sensitivity and specificity of the tests used. Patients with good periodontal health/gingivitis were more frequently found among those without OSA and less so among those with OSA, irrespective of their adherence to CPAP treatment. This points to an association between good periodontal health and normal breathing during sleep (i.e., nonexistence of OSA or CPAP treatment) on a patient level. While there is an apparent association between OSA and an increased risk of periodontitis, the nature and extent of this connection need further verification (Zhang et al., 2022).

In the present study, patients without OSA demonstrated a higher prevalence of periodontal health, yet no significant correlation was found across different stages of periodontal disease and OSA status. This could be attributed to our methodology of categorizing periodontitis into various stages, as significant differences were seen regarding bone loss. The criterion was either predominantly bone loss or no bone loss instead of four different stages. Consolidating data from the three periodontitis stages into a single group, the prevalence of periodontitis is comparable to that found in an epidemiological study in Jönköping (Norderyd et al., 2015; Wahlin et al., 2018). Interestingly, the present study shows a lower prevalence of periodontitis (i.e., merged periodontal groups) in those without OSA and a higher prevalence in those who were adherent to CPAP. The hypothesis that long‐term CPAP treatment decreases the risk for periodontal disease progression remains unverified, especially since the CPAP‐treated group had the lowest association with periodontal health or gingivitis, with only two such patients. However, in the adherent CPAP group, 12 out of 34 were classified as having periodontitis with BoP <10%, which may indicate a less active form of periodontal disease or possibly a historical condition. One possible reason could be the beneficial impact of CPAP treatment on OSA, although this observation was not statistically significant in our study.

Inflammatory markers such as cytokines for example, IL‐1β, IL‐6, which are elevated in saliva and gingival crevicular fluid, have been implicated in both periodontitis and OSA. These cytokines may play a role in activating osteoclastogenesis around the teeth, thereby increasing the risk of periodontitis (Téllez‐Corral et al., 2023). Additionally, alterations in oral microflora (Téllez‐Corral et al., 2022), potentially stemming from OSA‐induced oral dryness have also been hypothesized as a contributing factor (Lembo et al., 2021). The causal relationship between OSA and periodontitis is still debatable due to the diversity of study results and the heterogeneity of research methodologies, which makes a direct comparison difficult (Dort, 2017). Various studies define OSA using different criteria, ranging from questionnaire‐based assessments (Ahmad et al., 2013; Sales‐Peres et al., 2016) to more objective measures like the AHI (Gunaratnam et al., 2009; Latorre et al., 2018). Early studies including Gunaratnam et al. (2009) and Seo et al. (2013) did not categorize periodontal health/disease into different stages, primarily because they were conducted before the 2017 classification guidelines from the World Workshop of Chicago 2017 (Tonetti et al., 2018) were introduced. More recent studies, like Stazić et al. (2022), have started to categorize periodontitis patients according to the new classification. Another exception is Ahmad et al. (2013), which categorized patients similarly to this study and found a link between moderate to advanced periodontitis and OSA risk.

In the present study, a significant distinction was observed at the periodontal health/gingivitis stage, but this pattern was not evident in the more advanced stages of periodontal disease. Interestingly, the group of patients without OSA displayed a higher frequency of periodontal health compared to those with OSA. However, it is important to note that this non‐OSA group also included several individuals with advanced periodontal disease. Consequently, it is not possible to definitively conclude that the absence of OSA serves as a protective factor against periodontal disease. Rather, our findings indicate a higher prevalence of periodontal health or gingivitis in patients without OSA compared to those with the condition, and this trend holds true regardless of their adherence to CPAP treatment.

### 4.2 Limitations

Our study faced several limitations. The baseline data (2007–2009) primarily focused on OSA variables, lacking detailed information on oral health. This gap restricts our ability to track periodontal health changes over time. Additionally, the high initial age of participants and the long follow‐up period may have influenced the dropout rate, potentially skewing the results. Our 10‐year follow‐up study, designed with a broader oral health focus, was conducted around the time of the 2017 World Workshop on the Classification of Periodontal Conditions. While we made efforts to align our classifications with the new guidelines, some discrepancies remain. It would have been preferable to have apical X‐rays as a complement to bitewing pictures, which would have made it easier to follow the new classification more precisely. The decision to include bitewing pictures was decided before the new classifications were made and were mainly based on ethical reasons in form of less radiation. There was also practical reasons as both a dentist and a dental hygienist should be able to conduct the study. The modification to include bleeding in the stage formation was an active decision as it was necessary for the design of the study to know if the periodontal disease was in an active form or if periodontal bone destruction was due to a historical disease. Moreover, it was not possible to adjust for all concomitant factors such as hypertension, medication, initial AHI, or treatment with oral appliances. This is a common problem due to the median age and the size of the study population included.

### 4.3 Implications

Our findings underscore the need for integrated care approaches in managing patients with OSA and periodontal conditions. Early identification and multidisciplinary management are crucial for maintaining overall health. Dental clinicians should be vigilant about OSA symptoms and include comprehensive periodontal examinations for patients at risk.

### 4.4 Future research

Future studies should maintain the subgroup classification of OSA (i.e., based on severity levels using objective clinical OSA measures, such as AHI) and there should be larger, longitudinal studies to track periodontal health from baseline. Understanding the potential role of altered microbiomes in OSA and periodontal diseases, as well as the impact of CPAP treatment on inflammatory markers, remains fertile ground for future research. To be able to make comparisons between studies, a more homogeneous study design aligned with the new classification of periodontitis is important (Lembo et al., 2021).

## 5 CONCLUSION

Our study concludes that hypertensive patients with OSA do not exhibit significantly worse clinical periodontal status compared to those without OSA. However, the trend toward better periodontal health in individuals without OSA, irrespective of CPAP treatment, is an area for further investigation.

## AUTHOR CONTRIBUTIONS

Anders Broström started the project. Anders Broström, Henrik Jansson, Shariel Sayardoust, and Christine Kvarnvik had the main responsibility for the overall design of the study. Anders Broström (sleep), Hanna Ahonen, Malin Stensson (oral health), Shariel Sayardoust, Henrik Jansson, and Christine Kvarnvik (periodontal health) conceived ideas for the specific's parts in the current study, Christine Kvarnvik and Hanna Ahonen collected clinical oral health and periodontal health data. All authors have been part of the writing process.

## CONFLICT OF INTEREST STATEMENT

Saliva tests for examining levels of MMP‐8 in saliva (Periosafe, Dentognostics) were received without cost from Professor Timo Sorsa, Helsinki Finland.

## Data Availability

The data that support the findings of this study is available from the corresponding author upon reasonable request.
